# Deciphering the Interplay between Genetic Risk Scores and Lifestyle Factors on Individual Obesity Predisposition

**DOI:** 10.3390/nu16091296

**Published:** 2024-04-26

**Authors:** Danyel Chermon, Ruth Birk

**Affiliations:** Nutrition Department, Health Science Faculty, Ariel University, Ariel 40700, Israel; danyel.chermon@msmail.ariel.ac.il

**Keywords:** polygenic risk score, obesity, single nucleotide polymorphisms, physical activity, sugar-sweetened beverages, eating habits

## Abstract

Obesity’s variability is significantly influenced by the interplay between genetic and environmental factors. We aimed to integrate the combined impact of genetic risk score (GRS_BMI_) with physical activity (PA), sugar-sweetened beverages (SSB), wine intake, and eating habits score (EHS) on obesity predisposition risk. Adults’ (*n* = 5824) data were analyzed for common obesity-related single nucleotide polymorphisms and lifestyle habits. The weighted GRS_BMI_ was constructed and categorized into quartiles (Qs), and the adjusted multivariate logistic regression models examined the association of GRS_BMI_ with obesity (BMI ≥ 30) and lifestyle factors. GRS_BMI_ was significantly associated with obesity risk. Each GRS_BMI_ unit was associated with an increase of 3.06 BMI units (*p* ≤ 0.0001). PA markedly reduced obesity risk across GRS_BMI_ Qs. Inactive participants’ (≥90 min/week) mean BMI was higher in GRS_BMI_ Q3–Q4 compared to Q1 (*p* = 0.003 and *p* < 0.001, respectively). Scoring EHS ≥ median, SSBs (≥1 cup/day), and non-wine drinking were associated with higher BMI within all GRS_BMI_ Qs compared to EHS < median, non-SSBs, and non-wine drinkers. Mean BMI was higher in GRS_BMI_ Q4 compared to other quartiles (*p* < 0.0001) in non-wine drinkers and compared to Q1 for SSB’s consumers (*p* = 0.07). A higher GRS_BMI_ augmented the impact of lifestyle factors on obesity. The interplay between GRS_BMI_ and modifiable lifestyle factors provides a tailored personalized prevention and treatment for obesity management.

## 1. Introduction

The 21st century, the obesity epidemic presents a paramount challenge within the healthcare sector. This global epidemic transcends age and geography, impacting adults, adolescents, and children worldwide [1]. Obesity is not merely a condition of excess weight; it is a critical risk factor for a myriad of health complications, including but not limited to diabetes, cardiovascular diseases, hypertension, and certain cancer types, in both pediatric and adult populations [2]. The etiology of obesity is complex, with a substantial interplay between genetic predispositions and environmental factors [3]. Recently, the link between genetics and obesity has garnered considerable attention in scientific research. Central to this exploration are single-nucleotide polymorphisms (SNPs), which are variations in the DNA sequence that have been [4] linked to an increased propensity for obesity [5]. While each SNP individually exerts a modest effect on obesity risk, their collective impact is substantially more pronounced [6]. Modern genomic technologies have unveiled that the complexity of the human genome interplay in relation to obesity is greater than previously recognized, highlighting that sequence variations, including those in non-protein coding regions of DNA, and they are reshaping our comprehension of the genome’s role in obesity [7]. While certain genetic variants have been identified as predictors of susceptibility to obesity, it is becoming increasingly clear that obesity, like many other complex conditions, is characterized by a multifaceted genetic signature. The identification of genetic regions associated with obesity is predominantly conducted through Genome-Wide Association Studies (GWASs), which involve comparing the genetic sequences of individuals with obesity to non-obese, thereby pinpointing specific genetic variations linked to the condition [8]. To date, a multitude of SNPs associated with obesity have been identified [8,9]. Given the limited impact of each allele on obesity risk, a comprehensive scoring system known as the genetic risk score (GRS) has been developed, aggregating the cumulative effect of several obesity-related SNPs present in an individual, offering valuable insights for personalized prevention and treatment strategies [10]. Notably, the majority of GWAS studies have been conducted in Western populations, primarily focusing on European demographics [11]. However, there is a notable paucity of GWAS research encompassing other populations. This research aims to develop and validate a genetic model for obesity tailored to the Israeli population, thereby filling a critical gap in the current understanding of obesity genetics in this demographic. Obesity etiology involves genetic predisposition with an estimated 40–75% heritability combined with strong environmental factors [12]. Thus, genetic predisposition is exacerbated by an environment favorable to weight gain, marked by factors such as high-calorie foods, rapid eating, oversized food portions, sugar-sweetened beverages intake, excessive consumption of simple carbohydrates and sugars, physical inactivity, and sedentary behavior [13]. The interplay between GRS and lifestyle factors predisposing to obesity was scarcely studied. In our research, we delved into the intricate relationship between a multi-locus GRS, derived from a pre-analysis of individual SNPs, and key lifestyle factors such as PA, eating habits, and beverage consumption to illuminate the complex gene–lifestyle interactions and their influence on individual obesity predisposition. The current literature offers limited insights into the combined effects of GRS when considering lifestyle variables. Utilizing data from our comprehensive cohort, this research seeks to unravel the interplay between genetic predisposition and lifestyle choices, thereby contributing to the evolving field of personalized nutrition and interventions for obesity management.

## 2. Materials and Methods

### 2.1. Participants

The study is a cross-sectional analysis utilizing data from registry database (Lev-Hai Genetics LTD—MyGenes (Tel Aviv, Israel) listed in the registry database (#700068969)). Israeli adults (*n* = 5824) with a mean age of 55.78 ± 15.3 years were genotyped and completed an online lifestyle questionnaire (December 2021 to May 2023). The database was anonymous. Ethical approval for this study was obtained from the Ethics Committee of Ariel University (#AU-HEA-RB-20220214), and informed consent was obtained from all individual participants included in the study. Exclusion criteria included <18 years age; occurrence of a genetic disorder; or missing genetic, lifestyle, or anthropometric indices value (*n* = 100).

### 2.2. Anthropometric and Lifestyle Variables

Participants filled in an online questionnaire regarding their lifestyle and eating habits. Anthropometric measurements included height and weight. Height was reported in centimeters and weight in kilograms. BMI was calculated as the weight/(height)^2^ ratio (kg/m^2^). PA was computed by summing the weekly duration (minutes) of any PA the participants were engaging. The physically active group was defined as engaging in PA ≥ 90 min/week. Eating habits score (EHS) was calculated as the sum of Likert scale responses for a validated online eating habits questionnaire. The median (med) value of the EHS score served as the cutoff point; participants with an EHS score ≥ med were grouped as EHS ≥ med, while those who scored below the med were grouped as EHS < med. SSBs consumption was defined as consuming ≥ 1 cup/day. Wine consumption was categorized into non-drinkers and moderate wine consumers (≥1–3 drinks per week).

### 2.3. Genetic Risk Score (GRS_BMI_)

The candidate gene approach was employed to select relevant genes associated with obesity (BMI ≥ 30), with an emphasis on genes SNPs that demonstrated a significant association with obesity in previous GWAS studies [14]. To construct a comprehensive ‘weighted GRS_BMI_’, each SNP was weighted by its effect size (beta coefficient), on obesity risk, enhancing the predictive accuracy of the GRS_BMI_. SNPs were prioritized based on their minor allele frequency (MAF) (>0.01) in our population and their inclusion in the validated catalog of published GWASs. To ensure independent selection and tagging of SNPs, the HapMap database was utilized. SNPs with MAF exceeding 0.01 and tag SNPs with a pairwise linkage disequilibrium (r^2^) under 0.8 were chosen. In total, 86 SNPs associated with obesity were incorporated into the dataset. A stringent quality control was implemented. Additionally, SNPs with significant missing data were excluded to prevent biases. Further SNP selection was performed using a multivariate logistic model for obesity (BMI > 30 kg/m^2^) to identify each SNP under a significance threshold of *p* < 0.05, adjusted to sex, age, and type 2 diabetes mellitus (T2DM). The GRS_BMI_ was defined as the sum of the products of the number of risk alleles (0, 1, 2) of each SNP multiplied by its corresponding beta weight. The best-fit GRS model was constructed using eight SNPs: *TMEM* rs18939583, *ADRB3* rs4994, *FTO* rs9939609, *MC4R* rs2331841, *ADCY3* rs10182181, *BDNF* rs925946, *GIPR* rs11672660, and *BDNF* rs6265. For the analyses, participants were stratified into quartiles (Q1–Q4) based on their personal GRS_BMI_. Quartiles were defined by the 25th, 50th, and 75th of the GRS_BMI_ distribution. All analyses were conducted using the R-4.3.1 statistical software.

### 2.4. Statistical Analysis

The descriptive characteristics of the study participants were summarized using mean values and standard deviations for continuous variables, and median and interquartile range (IQR = 25th–75th percentile) for continuous variables not following a normal distribution. Frequencies and percentages were presented for the categorical variables. For continuous variables not following a normal distribution, the Mann–Whitney test was used to compare differences between participants with obesity and non-obesity groups. In the case of normally distributed continuous variables, an independent samples *t*-test was applied. For categorical variables, a Chi-square test was applied to determine the relation between obesity status and each categorical variable. To analyze the obesity risk across GRS_BMI_ quartiles and interactions with lifestyle, we conducted logistic regression models. Additionally, to understand the direct effect of genetic predisposition on BMI, we performed a linear regression analysis with BMI as the dependent variable and GRS_BMI_ as the independent variable.

## 3. Results

### 3.1. Participants

The study included a total of 5824 participants, with a mean age of 55.79 ± 15.3. Participants with obesity (BMI ≥ 30) had higher weight (*p* < 0.001), BMI (*p* < 0.001), and T2DM prevalence (*p* < 0.0001) compared to participants without obesity (BMI < 30). Concerning lifestyle factors, the BMI ≥ 30 group was less physically active with 14.9% meeting the PA ≥ 90 criteria compared to 26% in the BMI < 30 group (*p* < 0.0001), with a higher percent of SSB consumers (*p* < 0.0001) and lower percent of wine consumers (*p* < 0.0001) compared to participants with BMI < 30. Sex distribution varied significantly, with 72.39% of the BMI ≥30 group and 67.16% of the BMI < 30 group being females (*p* < 0.001; Table 1).

### 3.2. GRS_BMI_ and Obesity

**Figure 1:**
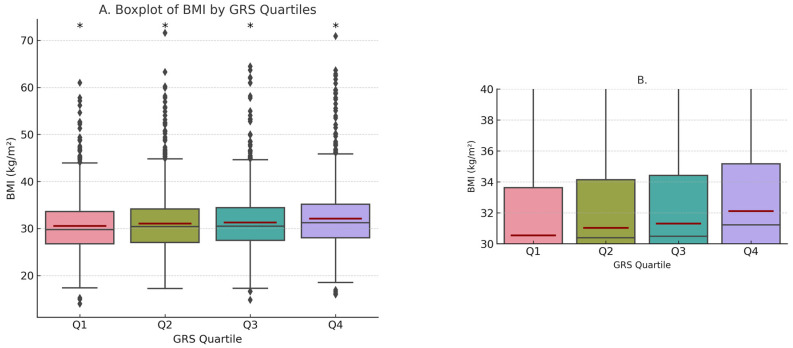
Boxplot of BMI across GRS_BMI_ Quartiles. (**A**) Median and mean BMI within each GRS_BMI_ quartile (Q1, Q2, Q3, Q4). Dark red line indicates the mean BMI. (**B**) Zoomed inset—enlarged view of mean and median BMI values for GRS_BMI_ quartiles. * Significance at the level of α < 0.001.

The GRS_BMI_ was significantly associated with obesity risk (OR = 2.72; 95% CI, 2.07–3.58; *p* < 0.001). Each additional GRS_BMI_ unit was associated with a 3.067 kg/m^2^ increase in BMI (R-squared = 0.02, *p* < 0.0001). As shown in Figure 1, higher GRS_BMI_ quartiles were significantly associated with obesity. Individuals in higher GRS_BMI_ quartiles exhibited higher BMIs, with a higher mean of 0.5, 0.8, and 1.6 BMI units for GRS_BMI_ Q2, Q3, and Q4, respectively, than GRS_BMI_ Q1 (*p* = 0.049, *p* < 0.001, and *p* < 0.001, respectively). The GRS_BMI_ Q4 mean BMI was significantly elevated by 1.1 and 0.8 BMI units compared to the mean BMI of Q2 and Q3, respectively (*p* < 0.001) (Figure 1).

### 3.3. GRS_BMI_ and Lifestyle Variables

#### 3.3.1. Physical Activity

Almost 20% of participants were physically active (≥90 min/week), while 80.05% were inactive or less active (<90 min/week; Table 2). Physically active participants had a lower obesity risk compared to physically inactive participants across all GRS_BMI_ quartiles (OR = 0.56, 95% CI = 0.43–0.72, and *p* < 0.0001; OR = 0.44, 95% CI = 0.34–0.57, and *p* < 0.0001; OR = 0.51, 95% CI = 0.4–0.67, and *p* < 0.0001; and OR = 0.48, 95% CI = 0.37–0.63, and *p* < 0.0001 for Q1, Q2, Q3, and Q4, respectively). Specifically, the GRS_BMI_ Q1 mean BMI for active participants was 29.0 ± 5.23, significantly lower by 1.9 BMI units than the mean BMI for inactive participants, i.e., 30.9 ± 5.85 (*p* < 0.0001). Active participants in Q2 had 2.6 units-lower mean BMI of 28.9 ± 4.73 compared to inactive participants (31.5 ± 6.17, *p* < 0.0001). The GRS_BMI_ Q3 mean BMI for active participants was 2.5 BMI units lower (29.3 ± 5.06) than the mean BMI for inactive participants (31.8 ± 6.09, *p* < 0.0001). Lastly, the GRS_BMI_ Q4 mean BMI for active participants was lower by 2.4 units (30.2 ± 5.66) compared to inactive participants (32.6 ± 6.43, *p* < 0.0001). For physically inactive participants, the results showed a significantly higher mean BMI in GRS_BMI_ Q4 compared to all other quartiles (*p* < 0.001, *p* < 0.0001, and *p* < 0.005 for Q1, Q2, and Q3, respectively), and in GRS_BMI_ Q3 compared to Q1 (*p* = 0.003; Table 2). This demonstrates a gradational increase in BMI from the lowest to the highest genetic risk-score quartiles among inactive participants.

#### 3.3.2. Eating Habits Score

An elevated EHS was associated with increased obesity risk across all GRS_BMI_ quartiles, adjusted for age, sex, and T2DM. Participants with EHS≥ med had a significantly higher obesity risk compared to those with EHS < med within GRS_BMI_ Q1, Q3, and Q4 (OR = 1.42, 95% CI = 1.15–1.75, and *p* = 0.001; OR = 1.51, 95% CI = 1.21–1.86, and *p* = 0.0002; and OR = 1.36, 95% CI = 1.09–1.69, and *p* = 0.006). The effect of EHS on BMI across all GRS_BMI_ quartiles analyses demonstrated notable differences. GRS_BMI_ Q1–Q4 participants with EHS ≥ med exhibited a 0.88–1.45-higher mean BMI units than those within the same GRS_BMI_ quartile with EHS < med (*p* = *p* < 0.001, *p* < 0.001, *p* < 0.0001, and *p* = 0.006, respectively). When comparing the mean BMI among individuals with EHS ≥ med, a higher BMI was noted in GRS_BMI_ Q4 and Q3 compared to Q1 (*p* < 0.0001 and *p* = 0.008, respectively), and for GRS_BMI_ Q4 compared to GRS_BMI_ Q2 (*p* = 0.02; Table 3).

#### 3.3.3. Sugar-Sweetened Beverages

Across each GRS_BMI_ quartile, SSB consumption was associated with an increased risk of obesity versus non-consumers (OR = 1.46, 1.48, 1.88, and 1.49 for Q1, Q2, Q3, and Q4, respectively). The mean BMI for SSB consumers was higher by 1.94, 1.55, 1.69, and 1.92 units within Q1, Q2, Q3, and Q4 than non-SSBs consumers (*p* = 0.0001, *p* = 0.002, *p* = 0.00001, and 0.0006, respectively). The GRS_BMI_ Q4 mean BMI was higher by 1.51 kg/m^2^ than GRS_BMI_ Q1 (*p* = 0.007), indicating an interaction between high GRS_BMI_ score and SSB consumption (Table 4).

#### 3.3.4. Wine

Moderate wine consumption showed a protective effect against obesity across all GRS_BMI_ quartiles adjusted for age, sex, and T2DM (Table 5). Specifically, GRS_BMI_ Q1, Q2, Q3, and Q4 moderate wine consumers had a 39%, 33%, 29%, and 35% lower obesity risk (OR = 0.61, 95% CI = 0.48–0.78, and *p* < 0.0001; OR = 0.67, 95% CI = 0.53–0.86, and *p* = 0.0013; OR = 0.71, 95% CI = 0.55–0.91, and *p* = 0.006; and OR = 0.65, 95% CI = 0.51–0.83, and *p* = 0.0006, respectively) compared to non-wine drinkers. GRS_BMI_ Q4 non-wine consumers’ mean BMI was higher compared to that of GRS_BMI_ Q1, Q2, and Q3 (*p* < 0.0001, *p* < 0.0001, and *p* = 0.005, respectively) non-wine consumers, and GRS_BMI_ Q3 was significantly higher than Q1 (*p* = 0.03).

## 4. Discussion

We developed a weighted GRS_BMI_ incorporating key SNPs to evaluate to evaluate obesity risk in the Israeli adult population. Our holistic approach was further enhanced by our unique integration of environmental factors, incorporating the important obesity etiology elements, underscoring the importance of population-customized genetic risk assessments in understanding and managing obesity. Our optimally calibrated GRS demonstrated a significant association with obesity (OR = 2.72; 95% CI, 2.07–3.58; *p* < 0.001) with a 3.067 kg/m^2^ higher BMI for each unit increase in GRS_BMI_ (*p* < 0.0001). Stratification into quartiles revealed significant average BMI escalation corresponding to the ascending GRS_BMI_ quartiles. Specifically, individuals in higher GRS_BMI_ quartiles exhibited higher BMIs, with a higher mean of 0.5, 0.8, and 1.6 BMI units than GRS_BMI_ Q1 for GRS_BMI_ Q2, GRS_BMI_ Q3, and GRS_BMI_ Q4, respectively. This combined pattern underscores the hypothesis that a higher number of risk alleles contribute to greater obesity predisposition. Consideration of both complex genetic predisposition and lifestyle factors highlights the potential of personalized treatment approaches to effectively target interventions with specific lifestyle-factor adjustments, thereby maximizing personalized health. The GRS_BMI_ included the SNPs TMEM rs18939583, ADRB3 rs4994, FTO rs9939609, MC4R rs2331841, ADCY3 rs10182181, BDNF rs925946, GIPR rs11672660, and BDNF rs6265, each of which has been previously linked to obesity [15] and was included in different GRS studies related to obesity predisposition in other populations, but in different SNPs composing the GRS. The *FTO* rs9939609 SNP was previously incorporated in GRS models predicting BMI and obesity across diverse populations, including African American and Caucasian populations [16], European adolescents [17], and the Iranian population [18]. Additionally, *BDNF* rs6265 and rs925946 have been incorporated into GRS association studies with obesity in European origin [6,19], and *BDNF* rs6265 among the Pakistani population [20]. *TMEM18* rs939583 was included in another GRS associated with BMI in individuals with extreme obesity compared to lean controls [21]. This inclusion of several SNPs in GRSs across diverse ethnic backgrounds highlights the versatility and global relevance of our GRS model in assessing obesity risk.

Our results indicate an interplay between GRS_BMI_ and environmental factors playing a significant role in the unexplained variability of obesity predisposition. Physical inactivity, consumption of SSBs, wine drinking, and disturbed eating behaviors increased obesity risk within all GRS_BMI_ quartiles, compared to PA, non-SSBs, or wine consumption and better eating behaviors. Combining a high GRS_BMI_ quartile with inactive lifestyle led to a significantly higher BMI. Moreover, participants who were physically less active (<90 min/week) or inactive had a significantly higher mean BMI compared to physically active participants independent of GRS_BMI_. In each GRS_BMI_ quartile being physically active had a significant protective effect for predisposition to higher BMI. Similarly, previous studies demonstrated that PA (different categorical levels) can significantly reduce the impact of GRS (different GRS composition) on BMI in subjects of European [22,23] and Han Chinese [24] ancestry. By setting a PA threshold of 90 min per week, our study acknowledges the dose–response relationship between PA and health outcomes, emphasizing that even a relatively low adherence to PA can mitigate the genetic predisposition to obesity. These results are inclusive for individuals with varying levels of PA, reflecting a broader spectrum of the population. It recognizes the challenges many individuals face in meeting the recommended levels of PA (e.g., 150 min of moderate-intensity activity per week) [25] and seeks to provide insights into the benefits of more achievable activity levels. This could be particularly important for sedentary individuals or those who find it difficult to allocate time for exercise due to busy schedules or other barriers, encouraging public health strategies that promote accessible changes. Across all GRS_BMI_ quartiles, individuals with favorable eating behavior score (EHS < med) exhibited a significantly lower BMI compared to those with less favorable eating behavior score (EHS ≥ med). Additionally, among participants with EHS ≥ med, those in the higher GRS_BMI_ quartiles (Q4 and Q3) were associated with higher BMIs compared to those in the lowest quartile (Q1). Early twin studies have shown a strong genetic influence on eating behaviors across all age groups, contributing to the increase in BMI [26]. However, only a few studies have investigated the role of eating behaviors in arbitrating the association between genetic predisposition to obesity. Using a unique EHS that encompassed a broad spectrum of common eating behaviors, we showed that, as the genetic risk for obesity increases, the impact of eating behaviors on BMI becomes more pronounced, highlighting the importance of favorable eating habits in individuals with a higher obesity genetic predisposition. This approach aligns with, yet distinctively differs from, the methodologies and findings of recent studies in this domain. A study involving adult participants from the Quebec Family Study indicated that genetic susceptibility to obesity (unweighted GRS) is partly mediated through undesirable eating-habit traits such as disinhibition and susceptibility to hunger [27]. Another study, one encompassing data from the GATE and the ALSPAC, found that the association between GRS_BMI_ and various eating behavior traits in relation to BMI was partially mediated by habitual, emotional, and situational disinhibition, as well as external and internal hunger [28]. Our results indicate that, with an escalating GRS_BMI_, the impact of these widely prevalent eating behaviors on BMI intensifies, underscoring the significance of considering everyday eating habits such as eating rate, portion size, and fast food, as well as emotional eating and late-night eating, in the broader population when assessing the interplay between genetic predisposition and obesity.

We demonstrated that the consumption of SSBs (≥1 cup/day) is associated with a significantly higher BMI compared to non-consumption within all GRS_BMI_ quartiles, and between SSBs consumers in the highest GRS_BMI_ quartile compared to the lowest (*p* = 0.007), amplifying obesity risk. SSBs are significant dietary source of added sugars and are a known contributor to obesity [29]. These results align with findings from other studies, such as those conducted on Swedish and US cohorts, which also reported a significant relationship between SSBs intake and BMI in individuals genetically predisposed to obesity [30,31]. Our study quantified a minimum of one serving of daily SSBs threshold consumption as a measurable variable, showing that, at this threshold, SSB consumption significantly affects BMI, especially in individuals with a higher GRS_BMI_, providing a practical framework for dietary recommendations and public health interventions.

To the best of our knowledge, this is the first study to incorporate the combined effect of GRS_BMI_ with wine consumption on obesity predisposition. Studies have shown an inverse association between wine and BMI [32,33,34]. We explored the relationship between moderate wine consumption across GRS_BMI_ quartiles and revealed a consistent pattern by which moderate wine drinkers exhibited a significantly lower risk of obesity compared to non-drinkers across all Q1-Q4 GRS_BMI_ quartiles (*p* < 0.0001, *p* = 0.0013, *p* = 0.006, and *p* = 0.006, respectively), with reduced BMI compared to non-drinkers (lower by 1.01, 1.28, 1.04, and 1.51 BMI units for Q1, Q2, Q3, and Q4, respectively). GRS_BMI_ Q4 non-wine drinkers’ BMI was significantly higher compared to that of GRS_BMI_ Q1, Q2, and Q3 (*p* < 0.0001, *p* < 0.0001, and *p* < 0.05, respectively, suggesting that moderate wine consumption might mitigate the impact of genetic risk on BMI, particularly in those with a higher genetic predisposition. The exact mechanisms by which alcohol intake affects body weight remain unclear and controversial. Moderate alcohol consumption has been shown to increase energy expenditure, with a more pronounced effect observed in women compared to men [35]. In addition, the role of resveratrol, a key wine polyphenol, has garnered attention for its potential effects on obesity [36,37]. Research has shown that resveratrol supplementation can mimic the effects of calorie restriction in humans with obesity, potentially improving metabolic profiles and insulin sensitivity. Resveratrol was also found to suppress postprandial glucagon in subjects with obesity, which could contribute to reduced glucose levels. Furthermore, resveratrol has demonstrated the ability to alleviate obesity-induced up-regulation of inflammatory cytokines and improve insulin signaling in adipose tissue [38,39,40]. Although moderate wine consumption was associated with a lower obesity risk in our cohort, this should not be interpreted as a causal relationship. Further studies are necessary to explore the underlying mechanisms, which may involve factors beyond the direct effect of wine consumption itself, and to determine whether these findings can be generalized to different populations or if they hold true under randomized controls conditions. The findings from this study underscore the importance of early identification of individuals at high genetic risk for obesity which enables targeted intervention. Public health initiatives can leverage these insights by integrating genetic risk assessments into existing health programs to tailor lifestyle modification and community-based activities designed to promote PA and healthy eating behavior among high-risk populations. Future research should focus on developing and refining specific implications and examining their effectiveness in diverse populations. While our findings advocate for the inclusion of genetic risk scores in developing personalized obesity interventions, they also underscore the necessity for further research to refine these tools and ensure their relevance across different populations.

Our study has its limitations, such as its cross-sectional design, which primarily allows for observational associations rather than causal inferences, coupled with the reliance on self-reported data obtained through a self-administered questionnaire. While self-reporting is a common methodological approach in large-scale epidemiological studies due to its cost-effectiveness and convenience, it is susceptible to various biases, including recall bias and social-desirability bias. While this method of data collection may not be as precise as other objective tools, it is important to note that the questionnaire employed in our study has undergone validation and demonstrated reliable performance in prior research [41,42]. This lends a degree of credibility to our findings, despite the inherent limitations of self-reported data. Consequently, our results may not be directly applicable to other demographic groups, such as younger individuals, rural populations, or those from different ethnic backgrounds. This limitation is crucial when considering the genetic components of obesity, as gene–environment interactions can vary significantly across different populations. Additionally, some potential confounding variables, such as socioeconomic status and dietary quality, were not available to us. Our study has several strengths, including a large population-based sample size. Additionally, a thorough assessment of genetic predisposition to heightened obesity risk was conducted, considering multiple variants known to increase susceptibility to obesity.

## 5. Conclusions

Our population-based study provides valuable insights into the interplay between genetic and environmental factors in the development of obesity. Our results emphasize the critical role of environmental factors to the general population and in particular to genetically predisposed to obesity sub-population with specific adjusted implications to manage and treat obesity, including engagement of ≥90 min/week in PA, avoidance of SSB consumption, pursuing recommended eating habits, and, if relevant, incorporating moderate wine consumption. Ideally, through genetic analysis, the identification of risk-predisposed obesity sub-populations will enable us to use specific environmental factors early to prevent the development of obesity and obesity co-morbidities. This study is essential for enhancing the applicability and relevance of genetic studies in clinical settings, ensuring that the benefits of genetic research are accessible and beneficial to a broader range of populations.

## Figures and Tables

**Table 1:** Descriptive characteristics of study participants.

Character	All (*n* = 5824)	BMI ≥ 30 (*n* = 3173)	BMI < 30 (*n* = 2651)	*p*-Value
Age (years)	55.78 ± 15.3	55.91 ± 15.4	55.63 ± 15.2	0.3
Height (cm)	166.42 ± 9.15	166.52 ± 9.4	166.29 ± 8.84	0.55
Weight (kg)	86.83 ± 19.6	98.13 ± 17.9	73.31 ± 11.2	<0.0001
BMI (kg/m^2^)	31.24 ± 6.06	35.28 ± 5.06	26.41 ± 2.66	<0.0001
Sex (female)	4050 (69.54)	1919 (72.39)	2131 (67.16)	<0.001
T2DM (*n*, %)	449 (7.7)	285 (8.98)	164 (6.19)	<0.0001
EHS score	11.60 ± 7.55	12.33 ± 7.68	10.73 ± 7.3	<0.0001
PA ≥ 90 (*n*, %)	1162 (19.95)	473 (14.9)	689 (26)	<0.0001
SSB consumers (*n*, %)	684 (11.74)	439 (13.84)	245 (9.24)	<0.0001
Wine consumers * (*n*, %)	1398 (24)	669 (21.08)	729 (27.5)	<0.0001

EHS, eating habits score; PA, physical activity; SSB, sugar-sweetened beverage (≥1 cup/day); wine consumers (≥1–3 drinks per week). * *n* = 146 missing data for wine.

**Table 2:** Obesity risk (BMI) among physically active ≥90 min/week and inactive (<90 min/week) participants across GRS_BMI_ quartiles *.

GRS_BMI_ Quartile	Mean BMI—Active (±SD)	Mean BMI—Inactive (±SD)	BMI Difference (Active vs. Inactive) (95% CI)	Obesity OR (Active vs. Inactive within Q) (95% CI)	Mean BMI between GRS_BMI_ Quartiles (Inactive) **
Q1 (*n* = 1465)	29.0 ± 5.23	30.9 ± 5.85	−1.9 (−2.56–(−1.2))	0.56 (0.43–0.72)	-
Q2 (*n* = 1456)	28.9 ± 4.73	31.5 ± 6.17	−2.6 (−3.25–(−1.95))	0.44 (0.34–0.57)	NS
Q3 (*n* = 1455)	29.3 ± 5.06	31.8 ± 6.09	−2.5 (−3.15–(−1.78))	0.51 (0.40–0.67)	0.003 ^a^
Q4 (*n* = 1448)	30.2 ± 5.66	32.6 ± 6.43	−2.4 (−3.14–(−1.64))	0.48 (0.37–0.63)	<0.001 ^a^ <0.0001 ^b^ <0.005 ^c^

GRS = genetic risk score; Q = quartile. * Adjusted for age, sex, and T2DM. ^a^ Compared to Q1; ^b^ compared to Q2; ^c^ compared to Q3. ** All *p*-values are Bonferroni-adjusted to control for multiple comparisons across quartiles.

**Table 3:** Obesity risk and BMI among EHS≥ and <median across GRS_BMI_ quartiles.

GRS_BMI_ Quartile (Q)	Mean BMI—EHS ≥ Median (±SD)	Mean BMI—EHS < Median (±SD)	BMI Difference (EHS ≥ Median vs. EHS < Median) (95% CI)	EHS ≥ Median vs. EHS < Median within Q OR (95% CI) *	BMI EHS ≥ between across GRS_BMI_ Quartiles **
Q1 (*n* = 1465)	31.02 ± 5.78	30.05 ± 5.74	+0.97 (0.38–1.56)	1.42 (1.15–1.75)	NS
Q2 (*n* = 1456)	31.56 ± 6.11	30.44 ± 5.83	+1.12 (0.51–1.73)	1.20 (0.97–1.48)	0.02 ^b^
Q3 (*n* = 1455)	32.02 ± 6.02	30.57 ± 5.86	+1.45 (0.84–2.06)	1.51 (1.21–1.86)	0.008 ^a^
Q4 (*n* = 1448)	32.51 ± 6.37	31.63 ± 6.31	+0.88 (0.23–1.55)	1.36 (1.09–1.69)	<0.0001 ^a^

EHS = eating habits score; GRS = genetic risk score; Q = quartile; NS = non-significant compared to other quartiles. * Adjusted for age, sex, and T2DM. ^a^ Compared to Q1; ^b^ compared to Q4. ** All *p*-values are Bonferroni-adjusted to control for multiple comparisons across quartiles.

**Table 4:** Obesity risk among SSBs consumers and non-consumers across GRS_BMI_ quartiles.

GRS_BMI_ Quartile (Q)	Mean BMI—SSB Consumers (±SD)	Mean BMI-Non-SSB Consumers (±SD)	BMI Difference (SSB vs. Non-SSB) (95% CI)	(SSB vs. Non-SSB within Q) OR (95% CI) *	Mean BMI between GRS Quartiles (SSB Consumer) **
Q1 (*n* = 1465)	32.30 ± 5.66	30.36 ± 5.77	+1.63 (0.69–2.57)	1.46 (1.05–2.04)	NS
Q2 (*n* = 1456)	32.4 ± 6.63	30.85 ± 5.89	+1.55 (0.49–2.58)	1.48 (1.07–2.05)	NS
Q3 (*n* = 1455)	32.78 ± 5.92	31.09 ± 5.97	+1.69 (0.77–2.64)	1.88 (1.36–2.63)	NS
Q4 (*n* = 1448)	33.87 ± 6.91	31.88 ± 6.25	+1.92 (0.78–2.98)	1.49 (1.05–2.1)	0.007 ^a^

GRS = genetic risk score; Q = quartile; SSB = sugar-sweetened beverage; NS = non-significant compared to other quartiles. * Adjusted for age, sex, and T2DM. ^a^ Compared to Q1. ** All *p*-values are Bonferroni-adjusted to control for multiple comparisons across quartiles.

**Table 5:** Obesity risk among moderate wine drinkers and non-drinkers across GRS_bmi_ quartiles.

GRS_BMI_ Quartile (Q)	Mean BMI—Wine Drinkers (±SD)	Mean BMI—Non-Drinkers (±SD)	BMI Difference (Drinkers vs. Non-Drinkers) (95% CI)	(Drinkers vs. Non-Drinkers within Q) OR (95% CI) *	Mean BMI between GRS_BMI_ Quartiles (Non-Drinkers) **
Q1 (*n* = 1424)	29.85 ± 5.79	30.86 ± 5.77	−1.01 (−1.7–(−0.31))	0.61 (0.48–0.78)	-
Q2 (*n* = 1423)	30.18 ± 4.85	31.46 ± 6.32	−1.28 (−1.91–(−0.66))	0.67 (0.53–0.86)	NS
Q3 (*n* = 1416)	30.58 ± 5.32	31.62 ± 6.16	−1.04 (−1.73–(−0.36))	0.71 (0.55–0.91)	0.03 ^a^
Q4 (*n* = 1415)	31.05 ± 5.35	32.56 ± 6.62	−1.51 (−2.2–(−0.83))	0.65 (0.51–0.83)	<0.0001 ^a^ <0.0001 ^b^ 0.005 ^c^

* Adjusted for age, sex, and T2DM. Physically active = PA ≥ 90 min/week. ^a^ Compared to Q1; ^b^ compared to Q2; ^c^ compared to Q3; NS = non-significant compared to other quartiles. ** All *p*-values are Bonferroni-adjusted to control for multiple comparisons across quartiles.

## Data Availability

The original contributions presented in the study are included in the article, further inquiries can be directed to the corresponding author.
